# Diagnosis of Occlusal Caries with Dynamic Slicing of 3D Optical Coherence Tomography Images

**DOI:** 10.3390/s20061659

**Published:** 2020-03-17

**Authors:** Minh N. Luong, Yasushi Shimada, Kazuyuki Araki, Masahiro Yoshiyama, Junji Tagami, Alireza Sadr

**Affiliations:** 1 Department of Restorative Dentistry, University of Washington, Seattle, WA 98195, USA; mndluong@uw.edu; 2 Department of Operative Dentistry, Graduate School of Medicine, Dentistry and Pharmaceutical Sciences, Okayama University, Okayama 700-8525, Japan; yoshiyam@md.okayama-u.ac.jp; 3 Division of Radiology, Department of Oral Diagnostic Sciences, Showa University School of Dentistry, Tokyo 145-8515, Japan; araki@dent.showa-u.ac.jp; 4 Department of Cariology and Operative Dentistry, Graduate School of Medical and Dental Sciences, Tokyo Medical and Dental University, Tokyo 113-8549, Japan; tagami.ope@tmd.ac.jp

**Keywords:** dentin, enamel, optical coherence tomography, radiograph, receiver operating characteristic (ROC) analysis, hidden caries, dentino-enamel junction DEJ

## Abstract

Detecting the extent of occlusal caries is a clinically important but challenging task required for treatment decision making. The aim of this study was to assess the diagnostic power of 3D swept-source optical coherence tomography (OCT) for evaluation of occlusal caries in comparison with X-ray radiography. Extracted human molars not exhibiting American Dental Association (ADA) criteria advanced caries were mounted in a silicone block and digital dental radiographs were captured from the buccal side. Subsequently, occlusal surfaces were scanned with a prototype Yoshida Dental OCT. Thirteen examiners evaluated the presence and extent of caries on radiographs and dynamically sliced 3D OCT video images, using a 4 level scale—0: intact; 1: enamel demineralization without cavitation; 2: enamel caries with cavitation; 3: dentin caries with or without cavitation. Sensitivity, specificity and area under operating characteristic curves (Az) were statistically analyzed (α = 0.05). Reliability analysis showed an excellent agreement among the 13 examiners for both methods. The OCT presented a significantly higher sensitivity and Az value for the detection of caries compared to radiographs (*p* < 0.05). Radiography showed especially low sensitivity for dentin caries (0–2 versus 3). Dynamic slicing of 3D OCT volumes is a powerful adjunct tool to visual inspection to diagnose the dentin occlusal caries in vitro.

## 1. Introduction

Dental caries is an infectious microbial disease of teeth resulting in local demineralization. Despite the tremendous reduction in the prevalence of smooth surface caries [1,2], occlusal caries still currently accounts for the majority of new lesions in the dentition of younger, post-fluoride generations [3]. In permanent dentition, occlusal caries involved 60% of total caries experience in children and adolescents. This could be attributed to the role of fluoridation. The reduction of smooth surface caries induced by water fluoridation was accompanied by the increase in the incidence of pit and fissure caries [4]. 

The frequent exposure to fluoridated products may reduce the rate of progression of caries and remineralize the outer enamel surface of carious lesion, masking an underlying extensive dentinal caries that is difficult to detect by visual examination only [5,6]. Therefore, this phenomenon is called hidden or occult caries or fluoride syndrome. These lesions are unopened when they show no cavitation observed by naked eyes. Although the etiology and presence of “hidden” caries is controversial [5,7], their significance is immense, because they could be neglected in low caries risk patients. Occlusal caries present a diagnostic challenge for dentists, as it is usually difficult to determine if the lesion has penetrated into dentin or not [5]. Indeed, bacterial penetration beyond the dentino-enamel junction (DEJ) and dentin involvement is considered a restorative threshold for treatment decision. When the non-cavitated lesion is confined to enamel, preventive treatment with monitoring is recommended [8]. However, once the lesion is cavitated and/or bacteria penetrate into dentin, a surgical approach should be considered [9]. 

In the concept of minimal intervention, caries management is based on early detection of lesions and remineralization of non-cavitated lesions with maximum preservation of tooth structure. Such criteria necessitate accurate and objective diagnostic tool of dental caries at an early stage [10]. To date, radiographic bite-wing images in combination with visual inspection is considered to be the gold standard procedure for caries detection. However, radiography has an intrinsic limit in sensitivity, because 30%–40% of minerals are already reduced before caries are radiographically evident [11]. The actual depth of penetration of carious lesion is hence deeper than that on a radiograph.

In a clinical routine, dentists usually rely on visual examination aided by radiograph to assess the lesion clinically. This examination is based on a lesion’s color, texture, and depth, sometimes aided by tactile sensation with a sharp explorer. However, using a sharp explorer for the detection of hidden occlusal caries can interfere with preventive methods, as it causes irreversible damage to enamel [12]. These methods are insensitive for detection at early stage. Therefore, the need for the development of a more advanced tool has been acknowledged by intensive research in recent years [13].

Optical coherence tomography (OCT) is an emerging modality in the current repertoire of available techniques, because it provides images of internal structure non-invasively. Based on the concept of low-coherence interferometry, OCT produces depth-resolved information about the scattering and reflection of light in the sample. Swept-source OCT uses laser source that emits different wavelengths sequentially in ultrahigh speed at a kilohertz rate to acquire real-time imaging with enhanced image resolution and 3D image construction. Optical coherence tomography has been used in the detection of carious lesion, cracks, and dental restoration defects [14,15,16,17,18,19]. Previous study validated early caries detection under OCT with histological and confocal laser scanning microscope (CLSM) [19]. 

It was reported that OCT cross-sectional images could detect natural fissure caries with a sensitivity and specificity superior to those of visual inspection [19]. However, with the nature of three-dimensional extension of caries, detection of occlusal caries using 3D OCT is highly desirable for the potential clinical application. Optical coherence tomography studies on caries have focused on various static 2D and 3D image analytic approaches [8,10,14,19]; however, the utility of dynamic slicing of 3D OCT data as a chairside method for diagnosis of caries has not been explored. Therefore, this study evaluated the accuracy of 3D OCT with dynamic slicing for detection of opened and unopened occlusal caries. The results of OCT were compared with radiographs of the lesion. The null hypothesis was that there was no difference in diagnostic power between OCT and X-ray radiography in occlusal caries at all levels.

## 2. Materials and Methods

### 2.1. Specimen Preparation 

Extracted human molars that had been stored at 4 °C in saline containing thymol to maintain hydration and prevent bacterial growth after extraction were investigated in this study. The usage of teeth was approved by the Institutional Review Board of Tokyo Medical and Dental University. The teeth were visually inspected by an experienced clinical dental practitioner (Y.S.) with 2.5× magnification loupes with headlights. Fifty-four teeth that did not have a visible restoration, crack, fracture, occlusal calculus, heavy discoloration or advanced caries lesion with exposed dentin were selected. The exclusion corresponded to American Dental Association (ADA) code advanced caries [1] or International Caries Detection and Assessment System (ICDAS) codes 5 and 6 [20]. The selected teeth were cleaned using a brush cone attached to low-speed handpiece with prophylaxis paste (Pressage, Shofu Inc., Kyoto, Japan), and mounted in 9 × 11 mm vinyl poly-siloxane silicone blocks (Exaflex Putty, GC Corporation, Tokyo, Japan) at the cervical level simulating a normal anatomic position. The blocks were used for ease of handling and to ensure reproducibility of OCT and radiographic imaging of the coronal tooth structure. Up to 3 locations on the occlusal pits and fissure on each tooth were randomly chosen and investigated. A total of 62 locations on 54 teeth were examined in this study.

### 2.2. Photography and X-ray Imaging

The occlusal surface of each tooth was photographed with a digital camera (Nikon D50, Nikon, Tokyo) with an AF-S Micro Nikkor 105 mm lens under standard photography conditions. Subsequently, digital radiographs were taken of each tooth with the X-ray unit targeting towards the buccal tooth surface and horizontal aspect positioned in the similar manner of clinical imaging with a 10 cm focus-film distance and an approximately 0 cm object film distance (resembling bitewing radiographs) using a digital dental radiograph device (Dentnavi Hands XD35, Yoshida Dental, Tokyo, Japan) operated at 60 kV and 7mA with an average exposure time of 0.6 s. The radiography was performed to simulate the presence of oral soft tissue using 1 cm thick water phantom.

### 2.3. Optical Coherence Tomography

**Figure 1:**
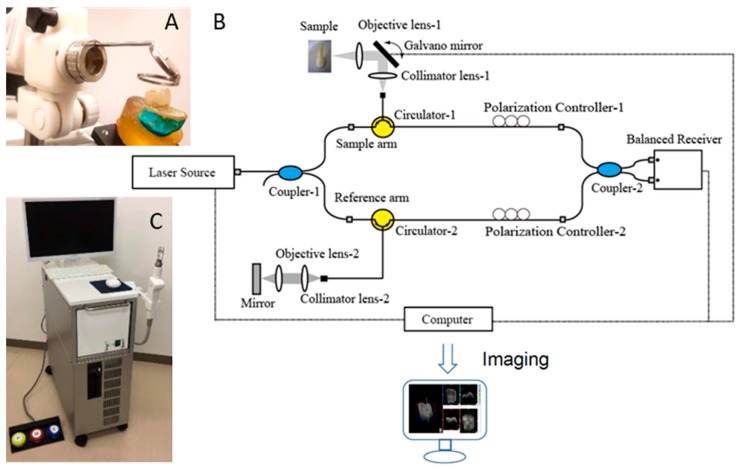
Prototype Yoshida Dental optical coherence tomography (OCT) system. (**A**) Specimen positioning to image the occlusal surface with the intraoral mirror tip for posterior teeth imaging attached to the handheld probe; (**B**) schematic diagram OCT system. An interference pattern is produced by splitting the beam of the Micro Electro Mechanical System (MEMS)-based laser source into two arms (i.e., reference arm and sample arm). The backscattered light from the sample is redirected back and recombined with the light from the reference arm in the coupler. The interference signal is transformed into raw A-scan data. Series of A-scans produce raw B-scan (2D) and the composition of 2D leads to the acquisition of 3D images; (**C**) overview of the OCT system with foot pedal for clinical imaging.

The prototype OCT system (Yoshida Dental OCT, Yoshida Dental Mfg, Tokyo, Japan) used in this study (Figure 1) constructs 3D images with a central wavelength of 1310 nm and a scan range of 140 nm at 50 KHz. The optical resolution in air was 11 µm in depth. A handheld scanning probe was used at a fixed distance over the tooth, with the scanning beam oriented 90 degrees to the occlusal surface. The full-resolution 3D image was obtained in 3.6 s over an occlusal field of view of 10 × 10 mm and optical depth of 8 mm to with 400 × 400 × 2048 voxel data.

### 2.4. Scoring by Examiners

The examiners who took part in this study included 13 dentists with a minimum of 5 years’ clinical experience as dental practitioners. In order to reach a consensus on the diagnostic criteria, the reference examiner (Y.S.) discussed the radiographic and OCT imaging methods in a 1 h session with examples that included OCT and photographic and radiographic images. For the calibration session, Y.S. used 10 extracted teeth images that were not included in the study.

After the discussion, the operators performed practical examination and scoring independently. 

The investigation sites were scored using a three-rank scale [14,17]:

0: Intact;
1: Enamel demineralization without cavitation;
2: Enamel caries with cavitation;
3: Open or unopened dentin caries with or without cavitation.

For X-ray scoring, the photographic image of the occlusal surface with the marked the investigation site was shown along with the radiograph of each tooth. These images were presented with a unique ID number and the examiners recorded their diagnosis with the ID number on a paper. Each image was screened for 20 s at the default brightness and contrast settings.

For OCT scoring, each 3D OCT data were presented as a video file with three panels: an occlusal view of the 3D reconstruction, an en face intensity projection image, and a mesio-distal B-scan that dynamically sliced back and forth in the bucco-lingual direction over 20–25 frames at the investigation site for 20 s at the default brightness and contrast settings using a custom-developed viewer software (KakumaViewer, Yoshida Dental Mfg). The occlusal photograph and OCT scan were presented with new ID numbers in a shuffled order to ensure that the examiners were blinded to the ID numbers of radiographs and photographs examined previously.

### 2.5. Validation of the Actual Scores

The diagnostic validation of actual scores was performed by histological examination. The specimens were sectioned along the tooth axis and trimmed from the buccal surface by wet silicon carbide papers under running water to reach the center of the investigation site represented in the OCT cross-sections followed by further polishing with diamond paste down to 3 µm. The polished surface was stained with 0.5% acid-red solution (Caries Detector, Kuraray, Medical Inc, Tokyo, Japan) for 10 s and rinsed under running water. The reference examiner performed the histological observation with a stereo microscope under the magnification levels of 3 ×–30 ×. The existence and appearance of a carious lesion at the investigation site and the penetration extent of dye was observed to determine the score following the criteria mentioned above. The number of locations inspected for each score were: score 0:15; score 1:14; score 2:22; score 3:11.

### 2.6. Statistical Analysis

Sensitivity and specificity indices for the detection of enamel and dentin caries using radiograph and OCT at 3 cut-off points (score 0 versus scores 1, 2, and 3; scores 0 and 1 versus 2 and 3; scores 0, 1, and 2 versus score 3) of 13 examiners were calculated [14,19]. The representative sensitivity, specificity and Az value obtained from receiver operating characteristic (ROC) curve in each group were calculated by averaging 13 examiners’ results in each category. The Az value (ROC area index) which is the area lying under the ROC curve. The sensitivity, specificity and Az values were compared between OCT and radiography using the Mann–Whitney U-test. To examine the reliability of each method, the interrater agreement for radiography and OCT was evaluated by intraclass correlation coefficient (ICC) in a two-way mixed-effects model with 95% confidence intervals (CIs). All data analysis was performed using statistical software SPSS 16 (SPSS, IBM, Chicago, IL) at a significance level of 0.05.

## 3. Results

**Figure 2:**
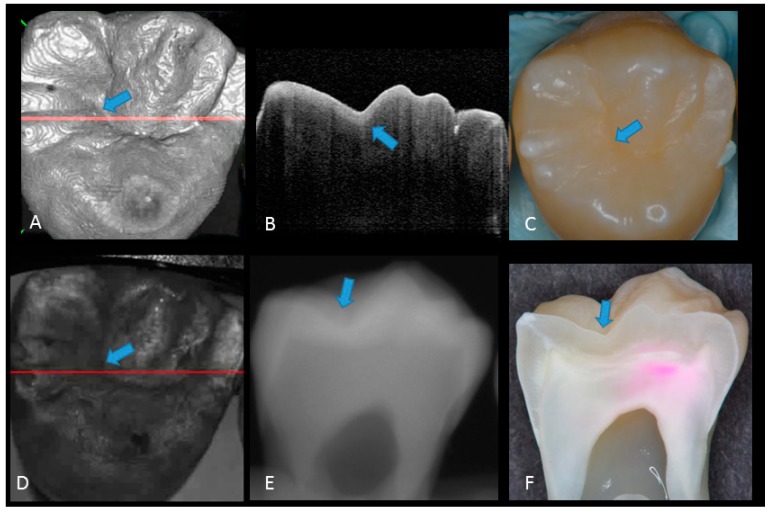
Sound enamel. (**A**) gray-scale three-dimensional (3D) OCT image; (**B**) cross-sectional OCT image along the red line in (A) and (D) with intact enamel with bright band across the fissure base extending over the cusps (arrow), suggesting a developmental feature (not acquired caries); (**C**) photographic presentation of occlusal enamel with seemingly intact fissure (arrow); (**D**) OCT en face intensity projection; (**E**) radiograph; (**F**) cross-sectional photograph with no dye at the observed fissure (arrow) confirming sound surface (score 0). The dynamic slicing 3D video is presented in Supplementary Materials: Video 1.

**Figure 3:**
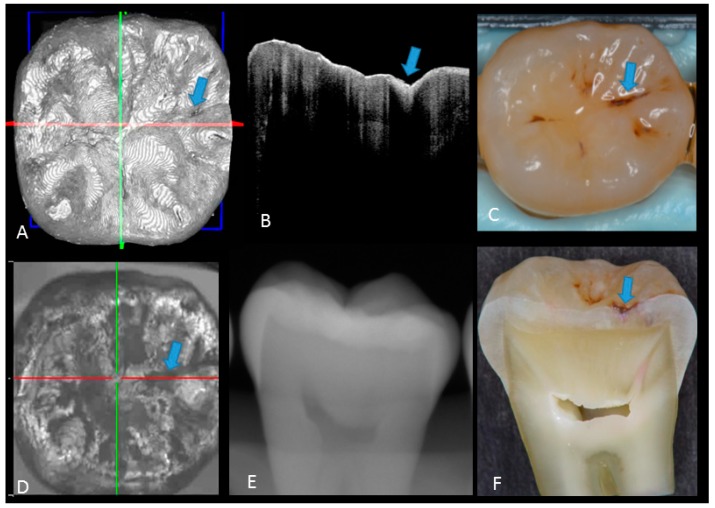
Enamel caries: (**A**) 3D OCT image; (**B**) cross-sectional OCT image along the red line in (A). Arrow shows a wedge-shaped area with increased scattering approximately half the thickness of the enamel, indicating demineralization with no surface breakdown; (**C**) photographic presentation of occlusal enamel with stained fissure (arrow); (**D**) OCT en face intensity projection; (**E**) radiograph showed no visible lesion; (**F**) cross-section view with shallow enamel dye penetration confirming demineralization without surface breakdown (score 1). The dynamic slicing 3D video is presented in Supplementary Materials: Video 2.

**Figure 4:**
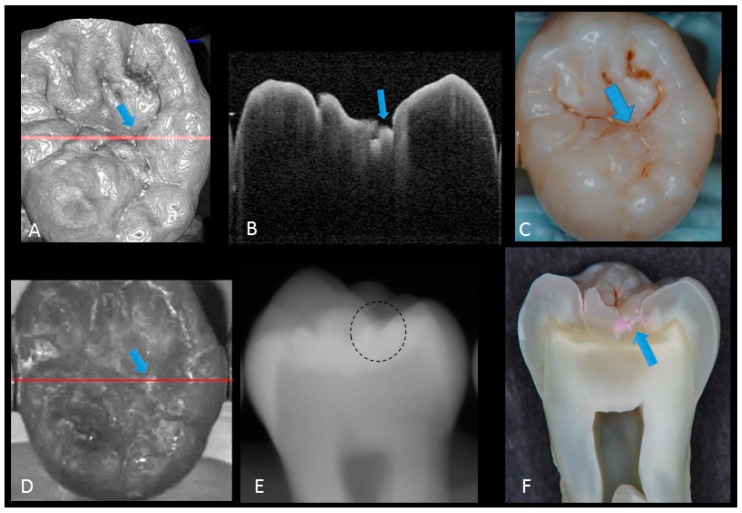
Enamel breakdown: (**A**) gray-scale three-dimensional (3D) OCT image; (**B**) cross-sectional OCT image along the red line in (A) and (D). Arrow shows cavitated caries; (**C**) visual score 2 of enamel cavitation; (**D**) OCT en face intensity projection; (**E**) enamel lesion was not distinguished on radiograph due to the superimposition of buccal and lingual cusps; (**F**) cross-section view with penetration of pink dye enclosed at enamel (score 2). Supplementary Materials: Video 3.

**Figure 5:**
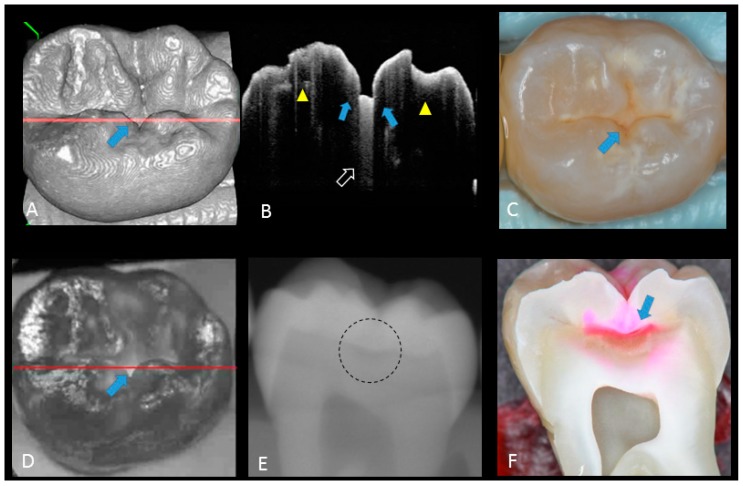
Hidden dentin caries; (**A**) 3D OCT image; (**B**) cross-sectional OCT along the red line in (A) and (D). Bold arrows show cavitated caries with discontinued enamel surface and scattering beyond dentino-enamel junction (DEJ) (triangle). Blank arrow suggests strong scattering from enamel into dentin (score 3); (**C**) photographic presentation of occlusal enamel; (**D**) OCT en face intensity projection; (**E**) dashed circle shows suspected radiolucency beyond DEJ on the radiograph; (**F**) cross-section view with penetration of red dye beyond DEJ, confirming the presence of caries infected dentin (score 3). The dynamic slicing 3D video is presented in Supplementary Materials: Video 5.

**Figure 6:**
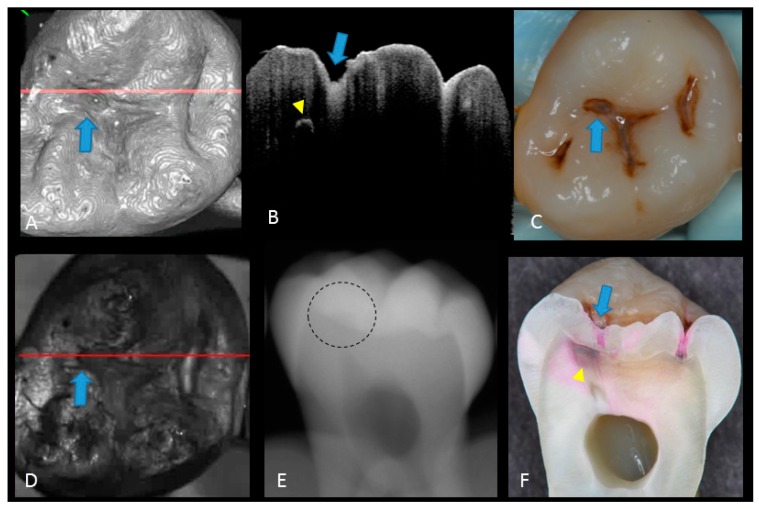
Dentin caries: (**A**) gray-scale three-dimensional (3D) OCT image; (**B**) cross-sectional OCT image of the lesion along the red line in (A) and (D). A separation of DEJ (triangle) was observed at the bottom at the lesion (arrow) indicating the lateral invasion of bacteria; (**C**) visual score 3 with visible dentin; (**D**) OCT en face intensity projection; (**E**) radiolucent lesion at DEJ; (**F**) cross-section view with penetration of dark red dye one-third of dentin thickness. The dynamic slicing 3D video is presented in Supplementary Materials: Video 4.

Representative photographic, radiographic and OCT images of each score are shown in Figure 2, Figure 3, Figure 4, Figure 5 and Figure 6. Representative dynamic slicing videos of the corresponding investigation sites, which were used for OCT scoring, are presented in the Supplementary Materials. In sound enamel (score 0; Figure 2, Video 1), the OCT images showed a consistent scattering over enamel surface without a significant, localized increase in scattering (brightness) at the enamel fissure base or any sign of surface breakdown. 

**Figure 7:**
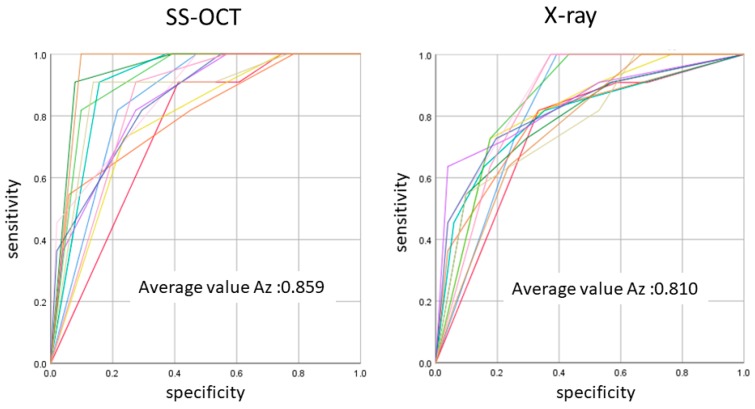
The average Az value of ROC with 13 lines represented by the Az value from thirteen examiners. There was a difference between OCT and radiography in the accuracy of detection in occlusal caries in three cutoff points (*p* < 0.05).

In initial enamel demineralization without cavitation (score 1; Figure 3, Video 2), a localized distinct increase in scattering was observed at the investigation site, usually at the base of an occlusal pit or fissure, without surface breakdown. In cavitated enamel caries (score 2; Figure 4, Video 3), the site exhibited cavitation, typically less than 1 mm in dimeter but no sign of strong scattering from underlying dentin. In dentin caries (score 3; Figure 5 and Figure 6, Videos 4 and 5), OCT showed dentin involvement with evidence of strong scattering beyond DEJ. Table 1 presents the sensitivity, specificity, and Az values for the detections of caries (0 versus 1–3), enamel breakdown (0–1 versus 2–3), and dentin caries (0–2 versus 3). The OCT showed significantly higher sensitivity for the detection of each caries level including enamel demineralization, unopened cavitated enamel caries, and dentin caries (scores 1–3: 0.946; 0.788; 0.741) compared to radiography (0.873, 0.643; 0.559). There was a significantly different sensitivity between OCT imaging and digital dental radiography (*p* < 0.05) (Figure 7) (Table 1). No significant difference was observed in comparison with digital dental radiography (*p* > 0.05) (Table 1). The Az values of OCT were also higher than those of radiography according to ROC analysis of 13 examiners’ results (Figure 7). A statistically significant difference was observed in ROC curve diagnostic power between the two methods (*p* < 0.05). An excellent ICC was observed for both systems, with OCT showing a coefficient of 0.963 (CI: 0.947–0.975) compared to 0.947 (CI: 0.925–0.965) for radiography.

## 4. Discussion

The results of the OCT in this study were compared with radiographs to evaluate the extension of lesion and validate the diagnostic potency of OCT. The focus was on two clinically important features on carious lesions, namely, enamel surface breakdown and dentin involvement. Sensitivity and specificity were used as measurements of accuracy in these two diagnostic tools at three cutoff points: 0 versus 1–3; 0–1 versus 2–3 and 0–2 versus 3. As a result, OCT examination presented a higher sensitivity for the detection of caries in all tested thresholds compared with radiographic examination, while there was no significance difference in specificity. Both methods presented a specificity superior to 0.8, which is a minimum value to assure a minimum false-positive fraction. It has been suggested that caries diagnostic methods should have value of at least 0.75 for sensitivity and over 0.85 for specificity [21]. The OCT imaging along with photographic observation seems to meet this threshold better than the combination of X-ray radiography with photographs, particularly for dentin caries (Table 1).

The ROC curve was also used for the comprehensive reflection of the diagnostic performance to each method, as it not only includes all cutoff points but also shows the relationship between sensitivity and specificity. The Az value provides a measure of performance across all possible thresholds. The high Az value represents the power of distinguishing positive and negative cases for the diagnostic system. In the current study, Az values confirmed the good performance of the OCT in detecting the presence and absence as well as extent of occlusal caries lesion. The OCT presented an average higher Az value compared to radiography in all cutoff points, corroborating its high sensitivity value.

In the selection of teeth for this study, advanced caries lesions based on the classification of the ADA and ICDAS were excluded. Following surface cleaning and under good lighting, these caries lesions present readily visible features such as large enamel surface breakdowns and remarkable visible dentin; therefore, their detection with visual inspection is less challenging compared with less advanced lesions. For non-cavitated dentin caries, the dentin lesion is covered with intact or incipient enamel lesions which could be misleading. Considering ICDAS classification criteria, the hidden caries is identified by code 4, presenting an underlying shadow in dentin with or without enamel breakdown [20]. A previous study comparing OCT with visual inspection (without radiographs) showed that the lowest sensitivity was observed at the dentin caries cutoff point for visual inspection [14]. A study comparing the validity of ICDAS from image-based and direct visual inspections also reported sensitivity values ranging 0.28 to 0.33 for caries requiring treatment as diagnostic threshold [22], highlighting the diagnostic challenge for these lesions. 

When in doubt, clinicians commonly use bitewing radiography to get further input for their diagnosis. However, the results of the current study suggest that even with radiographic help, these so-called “hidden” caries could still be missed by some of the experienced dentists, with a relatively low sensitivity of <0.55, while a higher sensitivity was obtained at score 0 (Table 1). This could be attributed to the superimposition of sound enamel that could obscure initial enamel changes, especially in cavitated shallow lesions confined to enamel or superficial dentin. In fact, radiographic examination could be subjective, depending on the visual perception of the examiner in addition to exposure to ionizing radiation. These false-positive and false-negative results have been previously reported for radiographic interpretation of occlusal caries [11,23]. Due to the superimposition of dense buccal and lingual cusps, non-cavitated dentin occlusal caries is difficult to see on radiography and only visible when there is a significant involvement of DEJ [24]. Previous studies concluded that detection of enamel lesions as score 1 and 2 with intraoral radiographs is less precise [25,26,27]. By the time an occlusal lesion is clearly visible on a bitewing radiograph, the depth of the lesion may have already penetrated the inner half of dentin [23]. This could explain the relatively good specificity of radiograph in detecting dentin caries at the cutoff point 0–2 versus 3 of this study. Also, lesions are usually underestimated in size or depth radiographically due to low attenuation of radiation in the lesion, physical properties of dental structure, or technical imaging mistakes. The result was in line with previously published studies illustrating that radiographs had high specificity and low sensitivity for dentin caries detection [28,29,30]. When compared with an adjunct laser fluorescence detection tool and visual examination, a study concluded that radiographic examination was the least accurate of the methods for diagnosis of enamel and dentin caries [31]. In addition, studies comparing the laser fluorescent and bitewing radiographs reported the smallest sensitivity for radiographs [25,32,33]. Therefore, the diagnostic accuracy of radiography alone for occlusal caries is questionable. Interestingly, the excellent agreement among the examiners based on the reliability analysis confirms the underlying limitation of radiographic interpretation for the diagnosis of this kind of caries regardless of the provider. It should be mentioned that the examiners visualized the radiographs at a standard brightness and contrast setting without image enhancements which could be considered as a limitation of the current study.

The potential of OCT to detect dental caries is based on detection of the backscattered light caused by submicrometer-size defects formed in demineralized lesions, leading to an increase in signal intensity which appears as bright regions in OCT images that can be readily distinguished from adjacent almost transparent sound enamel [8,10,14,17,18,19]. In the case of non-cavitated enamel demineralization as score 1, a subsurface zone with increased brightness compared with that in the surrounding smooth area on OCT. The loss of minerals at the lesion increases the porosity of enamel. Furthermore, OCT can detect the more advanced stage of dentin lesion when visual examination may be limited to enamel (Video 2). A unique feature of OCT imaging compared with both visual inspection and radiography is its ability to detect microstructural defects in the tooth; Figure 6 exhibited a clear separation at the DEJ, signifying the lateral spread of the bacteria at that transitional area and the deeper penetration of the lesion that seemed to be a minor (shallow) cavitation when being observed visually. From a clinical point of view, this is significant, since DEJ separation could also be associated with rapid advance of caries and symptoms such as hypersensitivity to cold and sweet substances. When caries progressed into cavitation, as score 2 in enamel caries, the scatterers will be completely removed as the surface collapses, resulting in appearance of a cavity with bright borders (Figure 4). The boundary of the lesion has not reached DEJ. When the caries penetrates further into dentin, a strong increase in brightness is seen as the remaining dentin collagen matrix being further demineralized and degraded, resulting in increased scattering and bright zones extending beyond the DEJ in grayscale OCT image [14].

The diagnostic power of OCT reported in the current study was remarkably higher than that reported previously [14]. This improvement could be attributed to the use of dynamic slicing of OCT images on the 3D volumes at the investigation site, which improves the detection of spatial features, such as the size of a cavitation and lateral spread at DEJ, compared with a single “best shot” B-scan image. This strategy demonstrated an excellent reliability among the 13 examiners, indicating its feasibility for clinical practice. Based on this observation, high-speed 3D scanning and reconstruction capability seems to be a necessity for a chair-side dental OCT system. It should be noted that the ROC curves showed a higher discrepancy among examiners and higher standard deviation for sensitivity at cutoff point 0–2 versus 3 for OCT, which indicates that the visual interpretation of OCT images requires appropriate calibration.

Another important limitation of the OCT imaging approach is that the imaging depth range could be limited to 2–3 mm or shallower depending on the attenuation of the near infrared light. While it seems that OCT is suitable for detection of occlusal caries, this modality may face limitations for deeper clinical applications, because the complex light-scattering in dentin is affected by the composition and structural orientation of this substrate [34,35].

The null hypothesis was partially rejected, as OCT could image every caries level with higher sensitivity compared to digital dental radiography. The sensitivity of OCT obtained from this study demonstrated that this modality can be used as an effective diagnostic tool to aid visual assessment. Especially, since it does not require any X-ray exposure and enable real-time visualization, it can serve as a safe option for a fast and precise diagnosis of caries extent.

## 5. Conclusions

Within the limitations of this study, dynamically sliced 3D OCT image was a powerful adjunct tool to visual inspection for diagnosing occlusal caries, particularly for dentin lesions with and without cavitation. In comparison to 2D OCT imaging, 3D dynamic slicing provided lateral extent of the lesion with an excellent reliability among the 13 examiners. Despite the difficulty of clinical diagnosis of hidden occlusal caries, OCT could enable the clinician to estimate the depth of the lesion in relation to the DEJ to indicate the necessity of operative intervention.

## Figures and Tables

**Table 1:** Cutoff values: a significant difference was observed in sensitivity and Az value receiver operating characteristic (ROC) curve between OCT and radiography (*p* < 0.05), whereas no such difference in specificity was shown. Different superscripts letters indicated significant difference.

Examiner	Methods	Sensitivity			Specificity			Az of ROC
		0 Versus 1–3	0–1 Versus 2–3	0–2 Versus 3	0 Versus 1–3	0–1 Versus 2–3	0–2 Versus 3	
1	OCT	1.000	0.788	0.818	0.667	0.667	0.769	0.849
	X-ray	0.917	0.697	0.727	0.600	0.700	0.731	0.819
2	OCT	0.979	0.939	0.909	0.800	0.633	0.596	0.751
	X-ray	0.896	0.909	0.818	0.800	0.633	0.654	0.745
3	OCT	0.917	0.788	0.909	0.933	0.833	0.923	0.943
	X-ray	0.833	0.545	0.545	1.000	0.800	0.885	0.788
4	OCT	0.958	0.667	0.545	0.600	0.667	0.942	0.802
	X-ray	0.875	0.576	0.364	0.800	0.767	0.962	0.787
5	OCT	1.000	0.939	0.727	0.867	0.700	0.769	0.782
	X-ray	1.000	0.818	0.727	0.800	0.633	0.808	0.813
6	OCT	0.917	0.697	0.909	0.867	0.767	0.846	0.905
	X-ray	0.521	0.394	0.455	0.867	0.933	0.942	0.798
7	OCT	1.000	0.788	0.455	0.667	0.667	0.981	0.862
	X-ray	1.000	0.697	0.455	0.333	0.733	0.942	0.879
8	OCT	0.833	0.636	0.364	1.000	0.933	0.962	0.845
	X-ray	0.792	0.545	0.636	1.000	0.900	0.962	0.836
9	OCT	0.958	0.879	0.818	1.000	0.933	0.904	0.915
	X-ray	0.917	0.788	0.727	0.867	0.733	0.808	0.853
10	OCT	0.771	0.636	0.364	0.867	0.900	0.981	0.848
	X-ray	0.833	0.424	0.455	1.000	0.867	0.962	0.818
11	OCT	1.000	0.848	0.909	0.733	0.600	0.731	0.837
	X-ray	0.917	0.727	0.636	1.000	0.767	0.827	0.844
12	OCT	0.979	0.879	0.909	0.733	0.700	0.865	0.879
	X-ray	0.896	0.818	0.545	0.867	0.667	0.885	0.781
13	OCT	0.979	0.758	1.000	1.000	0.933	0.885	0.951
	X-ray	0.958	0.424	0.182	1.000	0.833	0.942	0.764
Average (SD)	OCT	0.946 ^a^ (0.068)	0.788 ^c^ (0.102)	0.741 ^e^ (0.219)	0.826 ^g^ (0.132)^a^	0.764 ^h^ (0.120)	0.885 ^k^ (0.109)	0.859 ^m^ (0.057)
	X-ray	0.873 ^b^ (0.118)	0.643 ^d^ (0.163)	0.559 ^f^ (0.170)	0.841 ^g^ (0.186)	0.767 ^h^ (0.093)	0.870 ^k^ (0.095)	0.810 ^n^ (0.030)

Notes: Cutoff values: a significant difference was observed in sensitivity and area under receiver operating characteristic (ROC) curve (Az) between optical coherence tomography (OCT) and radiography (p< 0.05), whereas no such difference in specificity was shown. Different superscripts letters indicated significant difference.

## Supplementary Materials

The following are available online at https://www.mdpi.com/1424-8220/20/6/1659/s1; Video 1: Dynamic slicing of 3D SS-OCT scan for score 0; Video 2: Dynamic slicing of 3D SS-OCT scan for score 1; Video 3: Dynamic slicing of 3D SS-OCT scan for score 2; Video 4 and Video 5: Dynamic slicing of 3D SS-OCT scan for score 3.
